# Milk, Fresh from the Cow

**Published:** 1908-09-12

**Authors:** 


### Public Health and Hygiene.

### MILK, FRESH FROM THE COW.

It is generally admitted that the more dangerous pollutions of milk most frequently take place in the home of the consumer. This is inferred from the fact that the victims of zymotic diarrhœa, commonly accepted as a milk-borne disease, are found chiefly among the dirtier classes of the community, although the sources of milk for all classes are much the same. If these commonly accepted views be correct, they only serve to show the extraordinary impunity with which the public may consume disgusting quantities of filth in the disguise of milk. It is not practicable that health officers should personally supervise milking operations at each dairy farm throughout the country, and in the absence of a return from such inspections, it is difficult to estimate the standard of general cleanliness which attends this operation.

Certainly one would suppose that the practice at the milking competitions of agricultural shows is at least up to the general standard of care and cleanliness observed at the dairy farms not under direct and constant supervision. There are few who will not go further and affirm that a milking competition carried out before judges and in the public eye must exhibit a higher standard than might be looked for at the dairy farm under ordinary conditions. If this be so we leave the reader to judge what must be the composition of the liquid he consumes as milk, and with what degree of safety it may be prescribed for the delicate stomachs of infants and invalids in the light of the following description of a milking competition at an agricultural show. This account is embodied in a paper read by Dr. J. Howard-Jones, Medical Officer of Health of Newport, at the Congress of the Royal Sanitary Institute at Cardiff.

"The extent to which cleanliness is insisted upon by judges and practised by competitors at the leading agricultural shows affords a good illustration of the progress made in this country towards providing clean milk for the public. Competitors would naturally use every endeavour to favourably impress the judges under such conditions, and one would expect that those who are chosen to adjudicate in such competitions would insist upon: (1) Cleansing of the teats; (2) the wearing of clean linen overalls, etc., by competitors; (3) the cleansing of the hands; (4) the rejection of the first flow of milk."

He then describes what he actually observed while attending the daily milking competitions at one of the leading agricultural shows in the country:—

"The first day was devoted to competitions for women over eighteen years of age. With one exception the maids carried clean damp cloths for cleansing the cows' teats, but their ideas of bacterial cleanliness were certainly primitive. Only one maid rejected the first flow of milk. The others had evidently been brought up on strictly economical principles. After starting to milk, one maid coaxed the cow by patting its ungroomed flanks with her left hand, thus inoculating the milk with bacillus coli, which had there been preserved in 'dry culture' from time immemorial. Another one found that the cow's droppings soiled her pail, so the offending particles were first removed with the damp cloth from the outside of the pail and then from the inner surface, and finally her hands were further cleansed.

"The competition on the last day of the show was open to lads and lasses under eighteen years of age. An opportunity was thus afforded of judging the standard of cleanliness of the rising generation of milkers. Some of the maids did wear linen aprons or pinafores, and some provided themselves with clean damp cloths, but the lads scorned such unnecessary paraphernalia. Each competitor had a stool, but some of them were filthy, and none were clean. It is to be hoped that all competitors had recently washed their hands; certainly there was no opportunity of doing so within the arena, and unless the judges had occipital eyes they could not have seen whether a couple of late-comers had taken that precaution. One lad did wear a linen overall. Three of the maids went through the process of wiping the cow's teats; but one of them laid down the cloth on the well-used straw at her feet and carefully picked it up afterwards in order to clean the second cow's teats. Not one of the competitors rejected the first flow of milk. Why should they, when the results depended partly upon the quantity of milk submitted for the judges' inspection.

"One competitor was introduced to his second cow by a judge. With the assistance of his foot he made the cow "stand" to attention. The udder had just been resting upon an excellent culture of the organisms found in bovine dejecta. The first cow had not parted with her milk very readily, so the lad was in a hurry, and without any delay he forthwith started filling his pail—not with milk only though, for he had no time (?) to turn the deep end of the pail towards the posterior aspect of the cow on a certain occasion. Besides, he had noticed that the judge had weighed the contents of his first pail without apparently deducting anything for foreign matter. The crowd of onlookers were apparently either blind to these details or they recognised them as normal, for their whole attention seemed to be fixed upon the rate of the flow of milk into the pails, the weight produced, and the time taken by each competitor. The milk thus obtained was used at the model dairy, and was also sold at the official milk stall at the show."

It is unnecessary to comment on the filthy practices so naïvely disclosed. But it is certain that unless the British farmer awakens to the need of securing rudimentary conditions of cleanliness in the supply of fresh milk, the public will insist on being protected from the results of his grotesque incapacity. When a sufficient degree of disgust has been created in the public mind, the remedies likely to be proposed will be resented by the trade as vexatious and hampering, but the remedy at present lies in their own hands. Each fresh disclosure of the shortcomings of the milk vendor is diminishing his opportunities of reforming without outside interference.

